# Assessment by Ames test and comet assay of toxicity potential of polymer used to develop field-capable rapid-detection device to analyze environmental samples

**DOI:** 10.1007/s13204-014-0373-7

**Published:** 2014-11-02

**Authors:** Amanda Hebert, Michelle Bishop, Dhiman Bhattacharyya, Karen Gleason, Stephen Torosian

**Affiliations:** 1 US Food and Drug Administration, Winchester Engineering and Analytical Center, Winchester, MA 01890 USA; 2 US Food and Drug Administration, National Center for Toxicology Research, Jefferson, AR 72079 USA; 3 Department of Chemical Engineering, Massachusetts Institute of Technology, Cambridge, MA 02139 USA

**Keywords:** Biosensor, Cytotoxicity, oCVD, P(EDOT-co-3TE)

## Abstract

There is need for devices that decrease detection time of food-borne pathogens from days to real-time. In this study, a rapid-detection device is being developed and assessed for potential cytotoxicity. The device is comprised of melt-spun polypropylene coupons coated via oxidative chemical vapor deposition (oCVD) with 3,4-Ethylenedioxythiophene (EDOT), for conductivity and 3-Thiopheneethanol (3TE), allowing antibody attachment. The Ames test and comet assay have been used in this study to examine the toxicity potentials of EDOT, 3TE, and polymerized EDOT-co-3TE. For this study, *Salmonella typhimurium* strain TA1535 was used to assess the mutagenic potential of EDOT, 3TE and the copolymer. The average mutagenic potential of EDOT, 3TE and copolymer was calculated to be 0.86, 0.56, and 0.92, respectively. For mutagenic potential, on a scale from 0 to 1, close to 1 indicates low potential for toxicity, whereas a value of 0 indicates a high potential for toxicity. The comet assay is a single-cell gel electrophoresis technique that is widely used for this purpose. This assay measures toxicity based on the area or intensity of the comet-like shape that DNA fragments produce when DNA damage has occurred. Three cell lines were assessed; FRhK-4, BHK-21, and Vero cells. After averaging the results of all three strains, the tail intensity of the copolymer was 8.8 % and tail moment was 3.0, and is most similar to the untreated control, with average tail intensity of 5.7 % and tail moment of 1.7. The assays conducted in this study provide evidence that the copolymer is non-toxic to humans.

## Introduction

Presently, there is potential to drastically decrease the time it takes to carry out and confirm food pathogen assays. Utilizing a rapid-detection device could prevent outbreaks by confirming the presence of a pathogen prior to distribution or ingestion. Designing a real-time biosensor device to detect pathogens greatly decreases the chance of an outbreak, and even if an outbreak still occurs, this device could confirm the cause of the infection. Since the benefits of such a device are clear, a determination of possible toxicity effects created from its use should be determined.

Many food-borne pathogens are spread from surface contact, which makes environmental swabs an important part of hazard analysis critical control points (HACCP) at food manufacturing companies. Over 1,000 environmental swabs are used each year at just one large facility of a major United States food processer (Hood 2013). There are many food companies in the US alone that could significantly benefit from a real-time device that would provide rapid and highly sensitive results.

A prototype device has previously been generated that employed polypropylene microfibers coated with polypyrrole and functionalized with antibodies for *Escherichia coli* O157:H7 (McGraw et al. 2012b). The conductivity of the functionalized membrane was measured over time and could detect as few as 0–9 CFU/ml (McGraw et al. 2012b).

Desirable characteristics of such a device for detection of bacteria include being durable with high surface area microfiber material. Larger surface area allows greater attachment of biomolecules, which means this biosensor will have better sensitivity (Bhattacharyya et al. 2011, 2012; McGraw et al. 2012a). The melt-spun polypropylene will be coated on both sides with a very thin layer of conducting polymers, created from the monomers 3,4-ethylenedioxythiophene (EDOT) and 3-thiopheneethanol (3TE) on a microfibrous mat via oxidative chemical vapor deposition (oCVD). Iron(III) chloride is heated in a vacuum to allow deposition on the fibers. This is followed by heating the monomer mixture in a vacuum allowing single molecules to be polymerized from the iron(III) chloride deposited earlier.

After deposition, some of the samples were exposed to an acid rinse dopant exchange, in this study it was sulfuric acid rinse, then methanol, both of which are used to remove excess iron(III) chloride or monomers, which increases the conductivity and improves sensitivity (Bhattacharyya et al. 2011, 2012; Howden et al. 2013). By determining the toxicity potential of the chemicals in this study, it can be decided whether the polymerized copolymer (P(EDOT-co-3TE)) coating is safe for use or whether another composition would have less risk.

Some investigators looked at cell viability using phosphate buffered saline or polystyrene sulfonate for dopant exchange. None of these studies demonstrated significant change in viability. However, there are more subtle long-range effects that do not overtly express as loss of viability but may have effects via long-term exposure (Luo et al. 2008; Miriani et al. 2008; Asplund et al. 2009; Moral-Vico et al. 2014).

These viability studies were carried out on PEDOT created by electrodeposition. There have been no published in vitro toxicity studies on the chemicals used for nano-layer oCVD deposition nor on the copolymer P(EDOT-co-3TE). A tenet of nanomaterials is that they often take on diverse characteristic(s) peculiar to the nano state. It is prudent therefore to examine toxicity potential of such materials. If current chemical composition of the coating was determined to be cytotoxic or environmentally hazardous, other options to reduce cytotoxic effect could be explored. Among possible options would be modifying the EDOT to 3TE ratio, using 3-Thiophene acetic acid and polypyrrole (McGraw et al. 2012a) or other conductive copolymers.

The Ames test determines mutagenic potential of each test chemical by exposing them to mutated strains of *Salmonella typhimurium* that need histidine to survive. The *Salmonella* grows on minimal media and has a trace amount of histidine in a top agar. Either a phosphate buffer or a pooled rat liver S9 fraction that is mixed with co-factors is used for metabolic activation (Ames et al. 1973a, 1973b; Mortelmans and Zeiger 2000; Wessner et al. 2000; Hakura et al. 2003, 2005; Howden et al. 2013). The amount of S9 fraction that is added to the mix will greatly affect the amount of growth as well. Research on numerous known mutagens has demonstrated that they will have a higher count of colony forming units (CFU) than nonmutagenic chemicals tested with a strain (Hakura et al. 2005). If the percentage of S9 fraction in the mix is too low, there is no significant difference in the negative and positive controls (Forster et al. 1980). If the test chemical has the same heavy growth using various concentrations as the control mutagen, then the test chemical is considered to be mutagenic, and potentially carcinogenic.

When S9 mix is used, each strain uses the same known mutagen as a positive control. Using phosphate buffer instead of S9 mix means each strain will be tested with a different known mutagen as a control and compare against the test chemicals. The strain used in this study was TA1535 but other strains that are known to be tested in this assay include TA1537, TA1538, and TA98. Each has a different type of mutation. When TA1535 undergoes a reverse mutation, a base-pair substitution has occurred (Mortelmans and Zeiger 2000). By exposing the mutated strain to a known mutagen, there is a high amount of CFU since many reverse mutations occur. Without the mutagen, there are much fewer colonies due to the randomness of a mutation. A test chemical is considered a mutagen when the mutagenic frequency is above 2.0 or significantly lower than 1.0. This number is determined by dividing the mean CFU of the test chemical by the mean of the CFU for the untreated sample (Ames et al. 1973a, 1973b; Forster et al. 1980; Mortelmans and Zeiger 2000; Wessner et al. 2000; Hakura et al. 2003, 2005). By comparing the controls to the test chemicals, it can be determined whether they are potentially harmful.

The comet assay uses single-cell gel electrophoresis. A small amount of cells that are in agarose gel are lysed, the DNA unwinds, and then they undergo gel electrophoresis (Trevigen Inc. 2012). Once these cells are stained with SYBR green or SYBR gold, the comet-tail shape from the cell damage becomes apparent in epifluorescence microscopy and can be scored. Alkaline reagents are used because it is suggested that this is a more sensitive treatment that will detect single- and double-strand breaks (Speit and Hartmann 2006; Trevigen Inc. 2012). The larger the comet tail, the more DNA damage has occurred. After exposing cells to test chemical and there is little or no change in the cells, then the chemical is considered to have a very low chance of toxicity.

Numerous articles have discussed the comet assay, over 7,400 on PubMed alone, emphasizing its reliability and justifying the high frequency of use. The cell lines tested in this study are FRhK-4 and BHK-21. The BHK-21 cell line was also electroporated. Testing different cell lines derived from a variety of organisms aids in determining the potential for genotoxic affects of chemical exposure to kidneys (Genies et al. 2013). The three cell lines were from a variety of organisms and life stages. In this study, FRhK is fetal rhesus monkey kidney cells, BHK-21 is baby hamster kidney cells and Vero is African green monkey kidney cells. The cytotoxicity concerns for the chemicals used in this biosensor have been raised since there has been no previous studies addressing them in vitro.

For comprehensive comet analysis, software can be used to statistically analyze the data by viewing the slides using an epifluorescence microscope. It is important to consider how much of the original cell is intact and how much of the cell forms a comet (Olive et al. 1990a, 1990b; Olive and Banath 1995). Scoring the comets live with software gives highly accurate and quick results. When using comet assay IV software, several measurements can be instantly calculated and the results can be compared with the controls. It is critical to have these measurements when the cells look similar comparing various chemical treatments. It is important to consider how much of the original cell is intact and how much of the cell forms a comet. The high percentage of damaged cells indicates a great risk for toxicity.

After performing both Ames test and comet assay, the monomers show some potential of toxicity, but the deposited copolymer has a much lower potential, if at all. Based on current results, P(EDOT-co-3TE) coating has shown to be safe for use in the field and in food industries to prevent outbreaks.

## Materials and methods

### Ames test

*S. typhimurium* strain TA1535 was added to 3-ml overlay agar tube that consisted of agar, sodium chloride, and a histidine/biotin solution. Phosphate buffer pH 7.0 was made. To every negative control overlay tube, 60 µl of *S. typhimurium* and 500 µl of phosphate buffer were added (Mortelmans and Zeiger 2000; Wessner et al. 2000). Each positive control tube had buffer, *Salmonella* and 100 µg/ml concentration of sodium azide. The control and test chemicals were plated in triplicate. The concentration of EDOT was 1.33 g/ml, and concentration of 3TE was 1.144 g/ml were added to respective tubes. Deposited copolymer that was removed from a glass slide added to a microfuge tube containing glass beads and distilled water, then vortexed for 1 min at full speed. This created a relatively stable suspension of polymer with a range of sizes between 1 and 5 µm^2^, however most particles were 1 µm^2^ or less. There was 50 µl of the test solution added to each tube. The overlay was added to Davis minimal agar. After 5–7 days of incubation at 35 °C, the plates were counted and the mutagenic potential was determined.

### Removal of copolymer from glass slides

About twenty 500–750 µm diameter glass beads were added to a microfuge tube. Razor blades were sterilized and about 400 µl of electroporation buffer or 1X phosphate buffered saline (PBS) was placed on the slide before scraping the slide with a sterile razor blade to collect the sample. After the sample was added to the microfuge tube, it was vortexed for 1 min. The samples were placed at 4 °C for storage. The particles were analyzed using a confocal microscope and the most of the particle sizes ranged from 1 µm^2^ or less. One glass slide of copolymer was used per 400 µl of electroporation buffer. Since the thickness has been consistent with every deposition, this ensures that the same concentration of copolymer was used in each test.

### Electroporation

The procedure given in the electroporator manual was used. The voltage was 139 V, the amount of time 25 ms, the interval 0, the number 1, and the droop 9 %. After this procedure most of the copolymer particles have an average area of about 1 µm^2^ or less.

### CometAssay^®^

Each cell line was grown in Dulbecco’s modified eagle’s medium (ATCC). Some of the BHK-21 cells were electroporated. They are passaged regularly so they are not 100 % confluent when the comet assay is performed. The cells are removed from the flask first by rinsing with 37 °C 1X PBS (Sigma) two times, each time the cells were kept in the 5 % CO_2_ incubator for 1–2 min. Trypsin–Versene (Lonza) was added when it was time to remove the cells from the flask. The amount of time the cells were exposed to trypsin varies depending on the cell line. The alkaline protocol by Trevigen was followed. Lysis solution (Trevigen) was cooled at 4 °C prior to use. Approximately 1 × 10^5^ cells in 1X PBS (Sigma) of each cell type were prepared and can be found at ATCC. The cells were counted using a hemocytomer and Trypan blue (0.04 %). Cells were spun at 250×*g* for 9 min. Each slide was warmed in 37 °C incubator for at least 20 min to increase agarose adhesion. The LM agarose (Trevigen) was boiled in a beaker for about 5 min, then cooled in a 37 °C water bath for 20 min. Each sample had 25 µl of cells and 25 µl of a chemical. For every sample, there was 500 µl of LM agarose added to a 2 mL centrifuge tube. It was mixed gently by inversion, and then a 20 min treatment with the cells in LM agarose, hydrogen peroxide was kept at 4 °C and the other samples were kept in the 37 °C water bath for 20 min. After the 20 min treatment, two 50 µl samples were added to the CometSlide™ sample area. Cover slips were added to the agarose to keep the cells in a single plane. The slides were placed flat in the dark at 4 °C for 30 min. The slides were immersed in 4 °C lysis solution for 45 min. After draining the lysis solution, the freshly made alkaline unwinding solution was added, and the slides were immersed for 20 min at room temperature. About 850 ml of alkaline electrophoresis solution was added to the CometAssay^®^ ES unit, and the power supply was adjusted to 21 V. The electrophoresis solution was drained, and the slides were immersed in dH_2_O twice for 5 min, then 70 % EtOH for 5 min. The samples were dried at 37 °C for 30 min. The samples were stored in a desiccator at room temperature until they were ready for scoring. When it was time for scoring, 100 µl SYBR^®^ gold solution was added to each circle of dried agarose and stained at room temperature for 30 min in the dark. The slides were tapped and rinsed with water. The slides were then dried completely at 37 °C and then viewed by epifluorescence microscopy, (496/522 nm).The slides were scored using comet assay IV system.

## Results

**Fig. 1:**
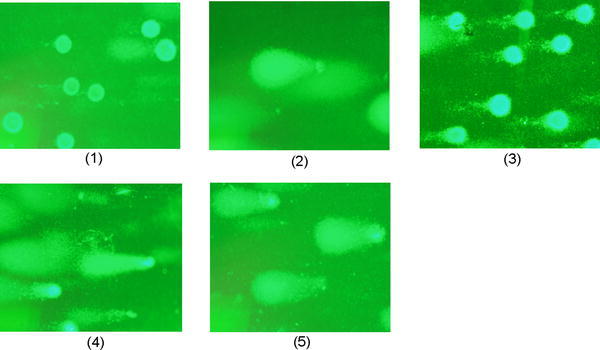
FRhK-4 cells. **1** Untreated, **2** hydrogen peroxide treatment, **3** P(EDOT-CO-3TE) treatment, **4** EDOT treatment, **5** 3TE treatment

**Fig. 2:**
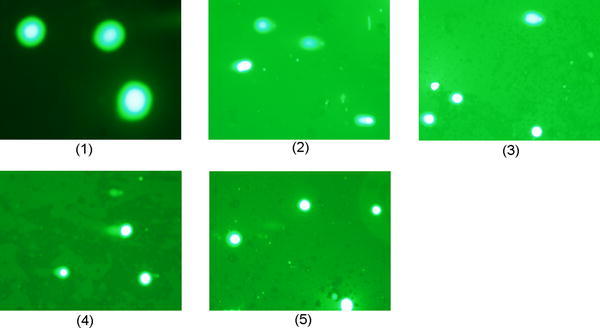
BHK-21 cells. **1** Untreated, **2** hydrogen peroxide treatment, **3** 3TE treatment, **4** EDOT treatment, **5** P(EDOT-co-3TE) treatment, **6** P(EDOT-co-3TE) injected into cells via electroporation, untreated

**Fig. 3:**
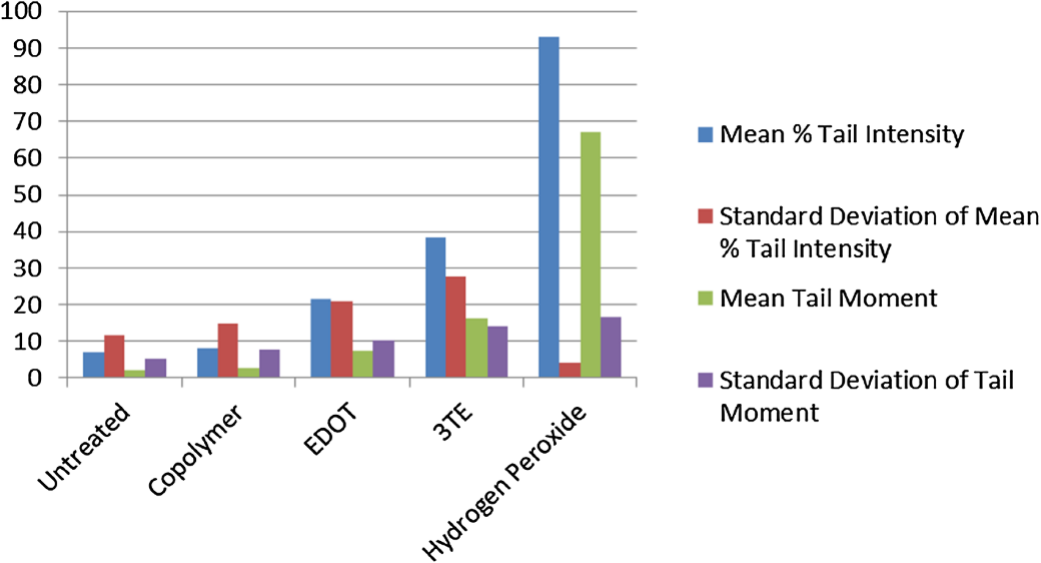
Percent tail intensity and tail moment for FRhK-4 cells

**Fig. 4:**
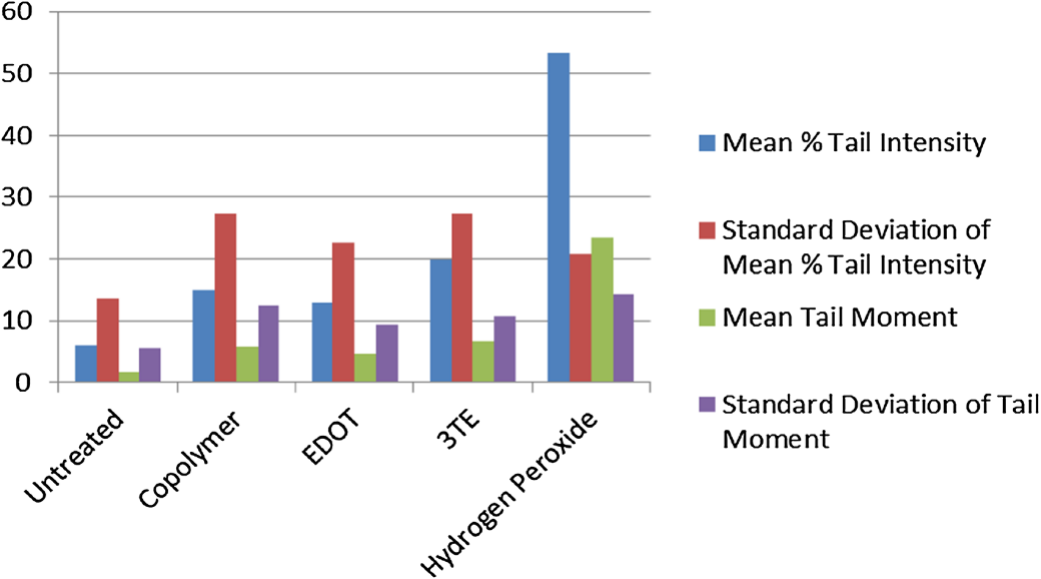
Percent tail intensity and tail moment for BHK-21 cells

**Fig. 5:**
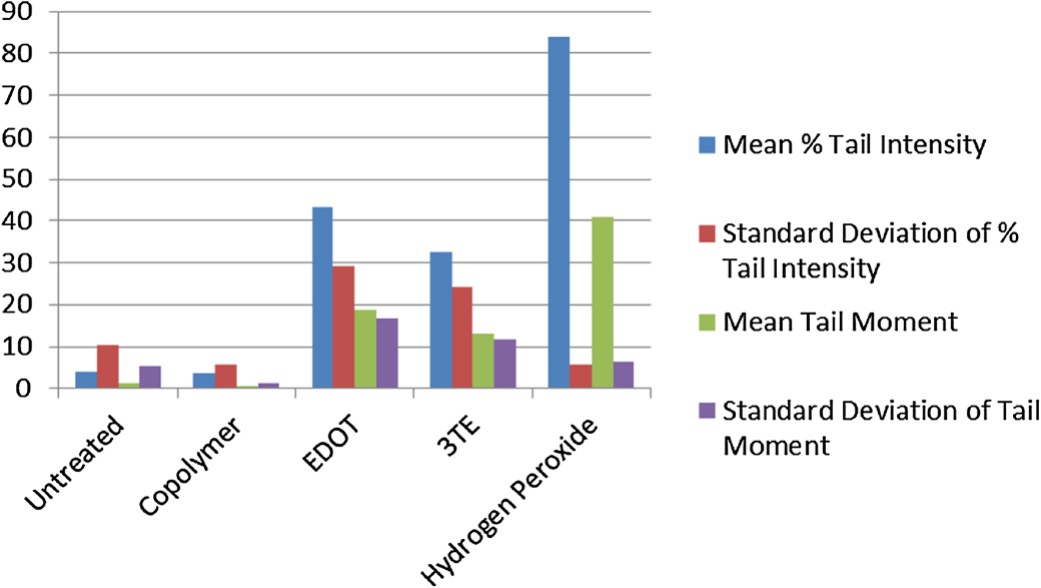
Percent tail intensity and tail moment for Vero cells*

**Fig. 6:**
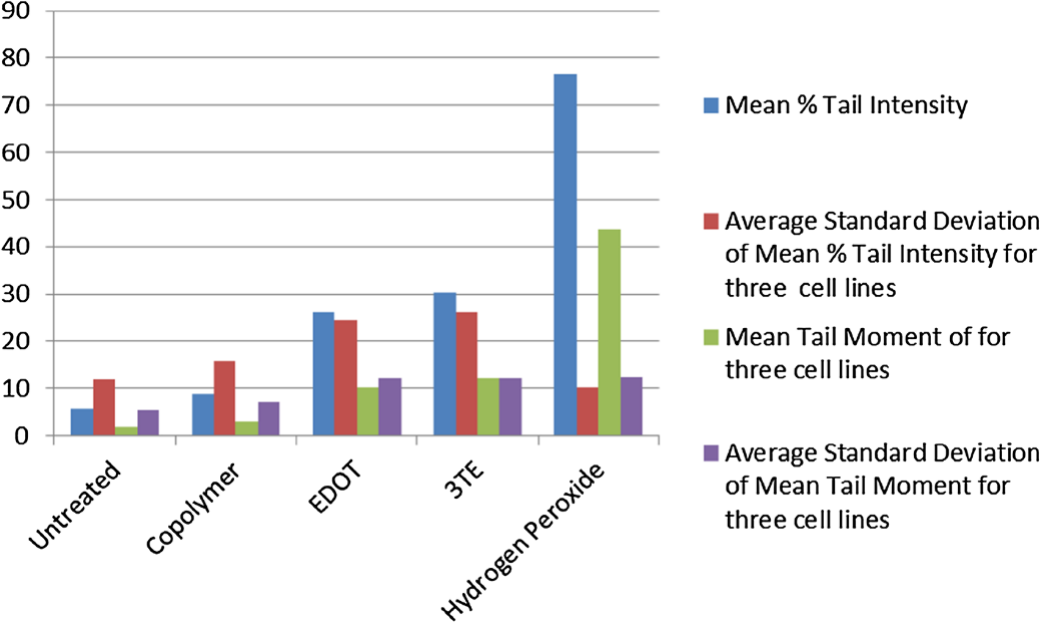
Average percent tail intensity and tail moment for three cell lines

Based on the strain *Salmonella* TA 1535 it is suggested that the polymerized copolymer, EDOT and 3TE have a mutagenic frequency below 1, meaning it has little to no potential for cytotoxicity (Table 1). However, 3TE has a mutagenic frequency of significantly lower than 1, at 0.56. The EDOT has a concentration of 1.33 g/ml and 3TE has a concentration of 1.14 g/ml (Figs. 1, 2, 3, 4, 5 and 6).

**Table 1:** Ames test

Chemical added	CFU/plate 1	CFU/plate 2	CFU/plate 3	Average	Mutagenic frequency
Sodium azide	102	157	131	130	2.6
None (just *Salmonella*)	47	59	44	50	1
Copolymer from glass slide	51	52	35	46	0.92
EDOT	35	62	33	43	0.86
3TE	42	27	15	28	0.56

Mutagenic frequency is measured by dividing the test chemical CFU by the negative control (*Salmonella*) CFU

The mean % tail intensity and tail moment are indications of toxicity. The tail moment is calculated using the tail length and the fraction of total DNA in the tail. Higher tail moment means there was damage to the cell after exposure to the chemicals. Different cell types were used to get a wider range of results because these cell tissues are sensitive to damage. The mean % tail intensity is determined based on the amount of DNA breaks in the tail relative to the head.

*Vero cell images look very similar to FRhK-4 and BHK-21 cells but are not included. The images are available on request.

## Discussion and conclusion

After the Ames test using strain *S. typhimurium* TA1535, the mutagenic frequency of 3TE was 0.56, which indicates toxicity potential because it is so much lower than 1. This suggests the chemical could be too toxic for the *Salmonella* to grow or the cells may have died after exposure. The mutagenic frequency of EDOT is 0.86, which shows it may be toxic since it is still lower than 1. The copolymer had a mutagenic frequency of 0.92, which indicates that mutagenicity potential is very low, if any at all, because this means the CFU that occurred after the copolymer treatment was very similar to the untreated CFU.

The mean of all cell types’ tail intensity and tail moment for the copolymer was 8.8 % and mean tail moment was 3, very close to the untreated control which had a mean tail intensity of 5.7 % and a mean tail moment of 1.7. The hydrogen peroxide was significantly higher than all the treatments, which resulted in a tail intensity of 76.7 % and tail moment of 43.8. Both the copolymer and untreated have much lower numbers than the EDOT which had 26 % with 10.3 tail moment and 3TE which had a 30.2 % tail intensity and the tail moment was 12. Based on these results, it shows that not only was the tail intensity percentage and tail moment less in the copolymer compared to other treatments, but the numbers are very close to the untreated control. It is important to note that BHK-21 cells that were exposed to the copolymer had slightly higher toxicity than EDOT, it is still significantly lower tail moment and tail intensity than the hydrogen peroxide control. The BHK-21 cells that were electroporated with the copolymer that were viewed in epifluorescence microscope did not have comet software analysis, but had been viewed in the epifluorescence microscope showed the vast majority of cells that were not damaged during the comet assay. Based on the overall results from comet assay and Ames test, the copolymer is considered to have low to no potential of toxicity.
